# Pediatric Intestinal Pseudo-Obstruction: An International Survey on Diagnostic and Management Strategies in the European Reference Network for Rare Inherited and Congenital Anomalies Intestinal Failure Teams

**DOI:** 10.1097/MPG.0000000000003788

**Published:** 2023-04-07

**Authors:** Annika Mutanen, Aysenur Demirok, Lucas Wessel, Merit Tabbers

**Affiliations:** From the * Pediatric Surgery, New Children’s Hospital, University of Helsinki and Helsinki University Hospital, Helsinki, Finland; the † Department of Pediatric Gastroenterology, Hepatology and Nutrition, Emma Children’s Hospital, Amsterdam UMC, University of Amsterdam, Amsterdam, The Netherlands; the ‡ Department of Pediatric Surgery, University Hospital Mannheim, University of Heidelberg, Mannheim, Germany.

**Keywords:** children, ERNICA, home parenteral nutrition, multidisciplinary team, parenteral nutrition, pediatric pseudo-obstruction syndrome

## Abstract

**Objectives:**

Pediatric intestinal pseudo-obstruction (PIPO) management is based on nutritional, medical, and surgical care while available evidence is scarce. The aim of this study was to outline the current diagnostic and management strategies in intestinal failure (IF) teams of the European Reference Network for rare Inherited and Congenital Anomalies (ERNICA) and to compare these practices to the latest PIPO international guidelines.

**Methods:**

An online survey on institutional diagnostic and management strategies of PIPO was conducted among the ERNICA IF teams.

**Results:**

In total, 11 of 21 ERNICA IF centers from 8 countries participated. On average, 64% of teams had ≥6 and 36% had 1–5 PIPO patients under active follow-up. In total, 80 of 102 PIPO patients were parenteral nutrition (PN) dependent while each IF team had median 4 (range 0–19) PN dependent PIPO patients under follow-up. On average, each center received 1–2 new PIPO patients per year. Diagnostics mostly followed current guidelines while medical and surgical management strategies were diverse.

**Conclusions:**

Numbers of PIPO patients are low and management strategies are diverse among ERNICA IF teams. To improve PIPO patient care, regional reference centers with specialized multidisciplinary IF teams and continuous collaboration across centers are needed.

**What Is Known**

- Pediatric intestinal pseudo-obstruction (PIPO) is a rare and most severe intestinal motility disorder which can lead to intestinal failure (IF).
- PIPO management is based on nutritional, medical, and surgical care while available evidence is scarce.

**What Is New**

- This is the first study on current diagnostic and management strategies on PIPO in IF teams of the European Reference Network for rare Inherited and Congenital Anomalies (ERNICA).
- Among ERNICA IF teams, numbers of PIPO patients are low and management strategies diverse.
- To improve PIPO patient care, regional reference centers with multidisciplinary teams and continuous collaboration across centers are needed.

Pediatric intestinal pseudo-obstruction (PIPO) is a rare heterogeneous group of most severe intestinal motility disorders which can lead to intestinal failure (IF). PIPO has an incidence of less than 1 in 40,000 to 100,000 (1–3). The diagnosis of PIPO is based on symptoms of chronic and recurrent obstructive symptoms with radiological features of dilated intestine with air-fluid levels in the absence of any occluding lesion (2,4). In PIPO, diagnostic challenges are common from the initial diagnosis to throughout life appearing recurrent ileus like episodes which often lead to delay in diagnostics, multiple operations, increased morbidity, and poor quality of life (5). The treatment of PIPO is challenging, associated with significant life-long morbidity and mortality and requires specialized multidisciplinary management (2,6). The goal of PIPO management is optimized IF treatment while avoiding complications and unnecessary operations (5,7). Furthermore, timely consideration for intestinal transplantation, the only curative treatment option for the majority, should be part of PIPO management strategies (2,5,7,8). In an European Society for Paediatric Gastroenterology, Hepatology and Nutrition (ESPGHAN) recommendation paper (2), specific diagnostic criteria and structured management approaches have been presented for PIPO while evidence behind the guidelines is still scarce. European Reference Network for rare Inherited and Congenital (digestive and gastrointestinal) Anomalies (ERNICA) is a network of expert multidisciplinary health care professionals from specialized health care providers across Europe, including 40 member hospitals in 12 European Union/European Economic Area countries. This network has the aim to pool together disease-specific expertise, knowledge, and resources to achieve health goals that may otherwise be unachievable in a single country. ERNICA IF working group gathers together professionals specialized in IF and PIPO management. To join the ERNICA IF working group, the center is required to meet general ERNICA center criterion and IF specific requirements for expertise (9).

The aim of the study was, first, to outline the current PIPO diagnostic and management strategies in ERNICA IF teams and, second, to compare clinical practice with the latest PIPO diagnostic and management recommendations of the guideline (2). In the future, results of this study may be used as baseline to establish more uniform and evidence-based diagnostic and treatment strategies in all ERNICA IF centers to improve PIPO patient care and outcomes.

## METHODS

An online survey was conducted between April 2020 and June 2022. After a pilot phase, the questionnaire was sent to all ERNICA IF group members and affiliated partner centers (n = 21), including centers in Austria (n = 1), Czech Republic (n = 1), Denmark (n = 2), Italy (n = 3), Finland (n = 1), France (n = 6), Germany (n = 2), The Netherlands (n = 2), Spain (n = 1), and Sweden (n = 2). The non-anonymous survey consisted of 49 closed questions on institutional diagnostic protocols and management of PIPO patients. The non-validated questionnaire was created by ERNICA PIPO working group members (AM, TMM, LW) based on ESPGHAN-led expert group consensus recommendation for PIPO (2). The full questionnaire is available as a Supplement, Supplemental Digital Content, http://links.lww.com/MPG/D124. Reminders were sent via email after the first invitation. If more than one professional answered the questionnaire from the same center, these answers were analyzed together to form one set of answers for each center. None of the filled questionnaires were removed because of missing data.

### Statistics

Categorical variables were summarized as frequencies and percentage and continuous variables as median and range. Data obtained from the survey was compared to the ESPGHAN-led expert group consensus recommendation for PIPO (2).

## RESULTS

### Participating ERNICA Centers

**FIGURE 1.**
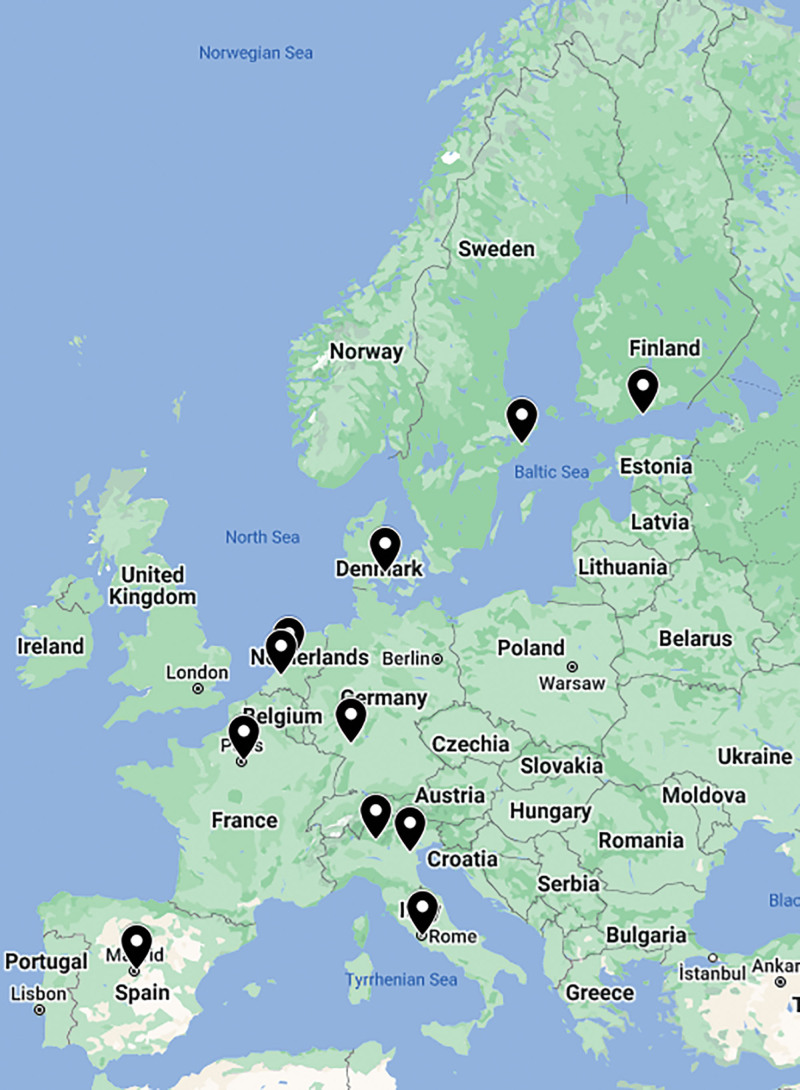
Eleven IF PIPO teams from 8 countries participated in the study, including Odense, Denmark; Helsinki, Finland; Paris, France; Mannheim, Germany; Padova, Italy; Bergamo, Italy; Rome, Italy; Amsterdam, the Netherlands; Rotterdam, the Netherlands; Madrid, Spain; and Stockholm, Sweden, representing 52% of the centers involved in ERNICA IF working group. ERNICA = European Reference Network for rare Inherited and Congenital Anomalies; IF = intestinal failure; PIPO = pediatric intestinal pseudo-obstruction.

In total, 11 ERNICA IF PIPO centers from 8 countries participated in the study (Odense, Denmark; Helsinki, Finland; Paris, France; Mannheim, Germany; Padova, Italy; Bergamo, Italy; Rome, Italy; Amsterdam, the Netherlands; Rotterdam, the Netherlands; Madrid, Spain; and Stockholm, Sweden), representing 52% of the centers involved in the ERNICA IF working group (Fig. 1). The questionnaire was filled in by 13 representatives from the participating centers, including pediatric gastroenterologists (n = 8), pediatric surgeons (n = 4), and pediatric anesthesiologist (n = 1). In 2 centers, the questionnaire was filled in by 2 representatives, including 1 surgeon and 1 gastroenterologist from each center. All non-responding centers (n = 10, 48%) were members or affiliated partners of ERNICA IF working group and tertiary reference centers for IF.

### The IF Teams and PIPO Patients

Overall, 102 PIPO patients were currently under follow-up in the 11 participating ERNICA IF centers. At the time of survey, 64% of teams had 6 or more (6–10 patients in 3 centers, 16–20 in 3 centers, and over 26 patients in 1 center) and 36% had 1–5 PIPO patients under follow-up. Each IF team had median 7 (range 0–23) PIPO patients under 16 years of age under follow-up. Altogether, 78% (80/102) of PIPO patients were currently parenteral nutrition (PN) dependent. Each IF team had median 4 patients (range 0–19) currently PN dependent. On average, each center received 1–2 new PIPO patients per year. All except one center continued follow-up after weaning off PN.

The IF teams treating PIPO patients covered median 2.5 million population (range 0.12–10 million). The upper age limit for follow-up was 18–19 years in most centers (n = 9) while 2 pediatric IF teams also offered follow-up for adult patients. All 11 participating centers had a multidisciplinary IF team responsible for PIPO patient care, consisting of pediatric gastroenterologist (100%), pediatric surgeon (100%), nurse (91%), dietician/nutritionist (82%), psychologist (82%), social worker (64%), genetic/metabolic medicine specialist (64%), urologist (55%), and general pediatrician/adolescent medicine specialist (36%). Most IF teams had specialized pediatric anesthesiologist, pathologist, and microbiologist available and participating in IF team meetings on request. All centers had a home parenteral nutrition team.

### PIPO Diagnostics

In all IF PIPO centers, the multidisciplinary team was responsible of making the diagnosis of PIPO. Routine diagnostic laboratory tests for PIPO, in order to exclude other underlying disease, included complete blood count, electrolytes, albumin, and renal and liver function tests by 100%, and inflammatory indices, thyroid function, and metabolic panel by 91%, and celiac serology by 82% of IF PIPO teams. Imaging studies were routinely used for diagnostics by all teams, most commonly abdominal radiography, small bowel follow-through, urinary tract ultrasound, and excretory urogram while other imaging studies were routinely less frequently used. Esophageal and/or anorectal manometry was routinely used by 55% and 45% of IF teams while antroduodenal and colonic manometry was used only by 27% and 18%. Upper gastrointestinal endoscopy and colonoscopy was done routinely for PIPO diagnostics by 73% of IF teams. All IF teams agreed that full-thickness intestinal biopsies are needed for PIPO diagnostics. Percentage of 91 recommended full thickness intestinal biopsies to be taken when therapeutic surgery is performed and 55% also for diagnostic purposes alone. Genetic testing was used routinely by all except 1 IF team while routine genetic panel was available only in 4 IF teams (Table 1).

**TABLE 1.** Studies used for PIPO diagnostics and evaluation by ERNICA IF teams (n = 11)

	Study routinely used	
	Yes, n	Yes, %
Imaging studies		
Abdominal radiography	11	100
Small bowel follow-through	11	100
Urinary tract ultrasound	10	91
Excretory urogram	7	64
Entero-magnetic resonance imaging (MRI)	5	45
Radio-opaque marker studies	4	36
Scintigraphy for measurement of gastric emptying	4	36
H_2_ breath test for measurement of small bowel transit	2	18
Entero-computed tomography (CT)	1	9
H_2_ (C13) breath test for indirect measurement of gastric emptying	1	9
Scintigraphy for measurement of small bowel and colon transit	1	9
Multidetector-row helical computed tomography (CT)	0	0
Manometry		
Esophageal manometry	6	55
Anorectal manometry	5	45
Antroduodenal manometry	3	27
Colonic manometry	2	18
Endoscopy		
Upper gastrointestinal endoscopy	8	73
Colonoscopy	8	73
Full-thickness intestinal biopsy		
When therapeutic surgery is performed	10	91
For diagnostic intent alone	6	55
No biopsies are needed	0	0
Laboratory tests		
Complete blood count	11	100
Electrolytes	11	100
Albumin	11	100
Renal and liver function tests	11	100
Inflammatory indices (ESR and CRP)	10	91
Thyroid function TSH, relates free hormone fractions	10	91
Metabolic panel (ammonia, lactate, urinary organic acids)	10	91
Celiac serology (tissue transglutaminase tTG, anti-endomysial IgA)	9	82
Fasting cortisol	8	73
Serum glucose and HbA1C	7	64
Cytomegalovirus	6	55
Ebstein-Barr virus	6	55
Connective tissue and skeletal muscle disorders (ANA, anti-ds-DNA, SCL-70, creatinine phosphokinase, aldolase)	6	55
Urinary porphyrins	4	36
Genetic testing		
Routinely used, yes/no	10	91
Routine genetic panel available, yes/no	4	36

Data are number of IF teams (n) and percentage.

ANA = Antinuclear Antibody; CRP = C-reactive protein; ERNICA = European Reference Network for rare Inherited and Congenital Anomalies; ESR = erythrocyte sedimentation rate; IF = intestinal failure; IgA = Immunoglobulin A; PIPO = pediatric intestinal pseudo-obstruction; TSH = thyroid stimulating hormone.

### PIPO Nutritional Management

According to the survey, nutritional management strategies favored oral feeding (100%), followed by optimizing enteral feeding without compromising intestinal function (62%), bolus feeding (46%), use of gastrostomy or jejunostomy feeds (31%), and continuous feeding (15%). The nutrition strategy was mostly guided by the multidisciplinary team (45%) or by the gastroenterologist and nutritionist together (45%) and rarely by a pediatric gastroenterologist alone (10%).

### PIPO Medical Management

Based on the survey, all of the prokinetic medications included in the survey were never or rarely routinely administered to all PIPO patients. Most commonly, medication for PIPO was used after individual medical planning, including amoxicillin/clavulanate, azithromycin, domperidone, erythromycin, metoclopramide, and octreotide (by 64%–82% of IF teams). Bethanecol and pyristigmine bromide were never used by 91% and 82% of the IF teams (Table 2).

**TABLE 2.** Medical management strategies of PIPO in ERNICA IF centers (n = 11)

	Routinely administered to all PIPO patients	Used after individual medical planning	Never used
Amoxicillin/clavulanate	1 (9%)	7 (64%)	3 (27%)
Azithromycin	0 (0%)	8 (73%)	3 (27%)
Bethanecol	0 (0%)	1 (9%)	10 (91%)
Cisapride	1 (9%)	2 (18%)	8 (73%)
Domperidone	1 (9%)	8 (73%)	2 (18%)
Erythromycin	1 (9%)	8 (73%)	2 (18%)
Metoclopramide	0 (0%)	8 (73%)	3 (27%)
Neostigmine	1 (9%)	3 (27%)	7 (64%)
Octreotide	0 (0%)	9 (82%)	2 (18%)
Prucalopride	2 (18%)	6 (55%)	3 (27%)
Pyristigmine bromide	1 (9%)	1 (9%)	9 (82%)

Data are number of participating centers with answer “yes” and percentage (%). Total number of participating ERNICA PIPO teams was 11.

ERNICA = European Reference Network for rare Inherited and Congenital Anomalies; IF = intestinal failure; PIPO = pediatric intestinal pseudo-obstruction.

### PIPO Surgical Management Strategies

The surgical management strategy for PIPO included considering (1) decompressing ileostomy by 82% and (2) venting and/or feeding gastrostomy and/or jejunostomy by 73% of the IF teams. The number of surgical interventions were minimized, firstly, in order to avoid potential complications (adhesions formation, prolonged paralytic ileus post-surgery) by 92% and, secondly, to avoid future obstructive episodes by 46% of IF teams. Small bowel resection was as much as possible avoided to (1) prevent occurrence of short bowel syndrome by 64%, (2) prevent occurrence of IF associated liver disease by 45%, (3) prevent reduction of abdominal domain in the view of potential future intestinal transplantation by 55%, but (4) not avoided by 36% of IF teams.

Intestinal transplantation was available in 4 of the 11 IF centers. Intestinal transplantation was considered as a therapeutic option for PIPO in case of (1) life threatening complications of parenteral nutrition (IF associated liver disease, loss of central line access) by 92% and (2) poor quality of life with high risk of morbidity and mortality (frequent pseudo-obstructive episodes necessitating repeated hospitalizations, difficult fluid-electrolyte imbalances, repeated septic episodes) by 8% of IF teams.

## DISCUSSION

To our knowledge, this is the first questionnaire on the current diagnostic and management strategies on PIPO in IF teams of ERNICA. The study aimed to gather baseline data on clinical practices in ERNICA IF centers and to compare these to the latest PIPO international guidelines. PIPO is a severe form of rare intestinal dysmotility disorders leading to IF and is related to significant morbidity and mortality (10). The rarity of the disease, a wide variety of subtypes and heterogeneity within PIPO patient group makes the management even more challenging (2,7). There is an existing guideline on PIPO management, including basis for multidisciplinary nutritional, medical, and surgical management while at the same time there is still lack of evidence (2). In this survey, we found a wide diversity in all aspects of PIPO diagnostics, management, and treatment strategies across ERNICA IF teams. We also recognized challenges related to the rarity of the disease, different management protocols, and availability of resources across ERNICA IF teams which gives us good background for future development of more uniform care for PIPO patients.

Multidisciplinary management of IF patients has been shown to improve patient outcomes (11). In this survey, we found that all participating ERNICA IF teams were multidisciplinary and had a home parenteral nutrition team, which likely has improved patient outcomes. Although this study gathered the ERNICA IF teams with most experience, the numbers of PIPO patients per center were low. This indicates the need for regional reference centers with specialized multidisciplinary teams to establish sufficient experience for the complex care that IF PIPO patients need. Furthermore, collaboration between specialized centers is essential for improving outcomes and creating guidelines for PIPO management. The advent in multidisciplinary care, parenteral nutrition, and intestinal transplantation allows more children with PIPO to survive into adulthood (11). These patients face a significant life-long risk for morbidity and are at need for continuous PIPO management and follow up (2,12,13). In this study, minority of ERNICA IF centers offered care for both children and adults. The collaboration between pediatric and adult IF teams and availability of multidisciplinary care after childhood remains to be a challenge. This should be one of the key future goals of improvement in PIPO management among ERNICA centers.

According to the ESPGHAN PIPO recommendation, PIPO diagnosis requires at least 2 out of 4 of the following: (1) objective measure of small intestinal neuromuscular involvement (manometry, histopathology, transit), (2) recurrent and/or persistently dilated loops off small intestine with air fluid levels, (3) genetic and/or metabolic abnormalities definitely associated with PIPO, and (4) inability to maintain adequate nutrition and/or growth on oral feeding (needing specialized enteral nutrition and/or PN) (2). In this study we did not seek to ascertain whether the PIPO diagnostic criteria were applied to individual patients and only studied overall diagnostic approach in each of the participating centers. For baseline diagnostic imaging studies for PIPO, a routine abdominal radiograph is recommended as a screening test for air-fluid levels and dilated bowel and a contrast study of the small intestine to exclude malrotation and organic lesions occluding the gut (2). When available, entero-magnetic resonance imaging (MRI) instead or in addition to contrast studies is recommended (2). The involvement of urinary tract is common in children with PIPO and therefore excretory urogram should be considered in all patients (2,14). In this study, the baseline diagnostics of PIPO included the recommended routine abdominal wall radiograph and contrast study of the small intestine in all IF teams. An entero-MRI was done routinely by less than half of IF teams, likely due to poor availability and need for anesthesia for young children. The urinary tract was examined routinely by most teams with urinary tract ultrasound and/or excretory urogram. Radio-opaque marker studies for the assessment of small bowel transit were done routinely by 36% of IF teams while the ESPGHAN expert group recommendation does not recommend the use of this study for PIPO diagnostics (2,13,15). Other recommended studies (2), including scintigraphy for measurement of gastric emptying or small bowel and colon transit, H_2_ breath test for measurement of small bowel transit, entero-computed tomography (CT), H_2_ (C13) breath test for indirect measurement of gastric emptying as well as multidetector-row helical CT, were rarely or never used routinely among the ERNICA IF teams. Overall, diagnostic imaging strategies for PIPO in ERNICA IF teams were mostly in line with the recommendations.

Manometric test provides both quantitative and qualitative assessment of esophageal, intestinal, colonic, and anorectal motor function by recording intraluminal pressure. The recommendation state that antroduodenal manometry should be performed in all children with a presumed diagnosis of PIPO in order to confirm the diagnosis, clarify the pathophysiology and optimize clinical management (2,16,17). However, in ERNICA IF teams, the antroduodenal manometry study was available and routinely performed in only 3 ERNICA IF centers. Other manometry studies, including anorectal manometry and esophageal manometry, were done routinely by half of IF teams. These results likely reflect the diversity in availability and required special expertise in manometric studies for PIPO diagnostics among ERNICA IF centers.

Although histopathological analysis and diagnosis rarely affect directly clinical treatment of PIPO, they provide information on prognosis and may direct future investigations. According to recommendations, histopathological analysis of intestinal biopsies should be performed in centers with expertise to undertake a full panel of neuromuscular labeling techniques (2). In this study, all ERNICA IF teams agreed that full-thickness intestinal biopsies are needed as part of diagnostics of PIPO. Out of all teams, 91% took routine biopsies when therapeutic surgery was performed and surprisingly only 55% for diagnostic purposes only. Although all IF teams agreed that full-thickness intestinal biopsies are needed for PIPO diagnostics, the use of biopsies for diagnostic purposes only was not uniform and lower than expected. This may be due to the threshold for invasive diagnostic testing in pediatric patients and availability of expertise in histopathological analysis.

Laboratory tests are useful in evaluating secondary forms of PIPO, related to systemic disease, some of which can be potentially curable. Therefore, baseline diagnostics of PIPO should include general laboratory tests for blood count, liver and renal function, inflammatory indices, and thyroid function in all cases and additional test based on clinical suspicion (ie, viral disease, diabetes mellitus, celiac disease, connective tissue, and skeletal muscle disorders) (2,18,19). In ERNICA IF centers, the baseline PIPO laboratory diagnostics fully followed the recommendations. However, there was more variation in additional diagnostic laboratory studies as on average 6 out of 11 teams were taking these tests routinely. Although most cases of PIPO are sporadic with only few known genetic forms, a genetic counseling is recommended at least in all cases of PIPO associated with other congenital abnormalities and patients with syndromic forms (2). Primary PIPO, including sporadic or familial myopathy, neuropathy, mesenchymopathy, mitochondrial diseases, or neuropathy, has been linked to mutations such as *ACTG2*, *SOX10*, *POLGI*, *FLNA*, *L1CAM*, *MYH11*, *MYLK*, *LMOD1*, *MYL9*, *RET*, *TYMP*, *RAD21*, and *SGOL1* (20–22). In ERNICA IF teams, genetic testing was in routine use in all except 1 center while routine genetic test panel was available in only 4 centers. This highlights the importance of future collaboration between specialized teams for research and to create uniform way for genetic testing in PIPO.

The medical management strategies of PIPO patients may include control of intestinal inflammation and bacterial overgrowth as well as promoting gastrointestinal motility (15,23). There is very limited evidence for the safety and efficacy of different medications for PIPO (2). Overall, in this study, great variability existed in PIPO medical management strategies as prokinetic medications were never used by some IF teams while others prescribed these to all PIPO patients. These results highlight the lack of evidence and need for collaborative studies and recommendations for medical management strategies for PIPO.

Decompressing ileostomy and venting and/or feeding gastrostomy and/or jejunostomy were considered by a majority of ERNICA IF teams while ESPGHAN expert group recommends considering these for all PIPO patients. Of ERNICA IF teams, 64% stated that small bowel resection was as much as possible avoided in PIPO in order to prevent short bowel syndrome, occurrence of IF associated liver disease, and/or reduction of abdominal domain in the view of potential future intestinal transplantation. In contrast, 36% IF teams reported to have lower threshold for small bowel resection. Overall, surgical management strategies were diverse across ERNICA IF teams likely reflecting the lack of evidence for different surgical approaches.

Availability of intestinal transplantation was not equal across IF teams as only 4 out of 11 centers had intestinal transplantation available in their center. Intestinal transplantation was considered for patients with PIPO according to recommendations in all IF teams, as intestinal transplantation indications were life-threatening complications of parenteral nutrition and poor quality of life with high risk of morbidity and mortality (2,24,25).

The strengths of this study include that this is the first survey on PIPO diagnostic and management strategies among ERNICA IF teams. Furthermore, the survey covered expert centers throughout Europe with large experience and relatively high study population on this rare disease.

The limitations of this study include a response rate of 52% and the low number of professionals (1–2) per team answering the questionnaire. It may be possible that centers not answering the questionnaire have no PIPO patients under follow-up. However, the exact reason other centers did not respond remain unknown which may lead to bias. Another important limitation is the lack of a validated questionnaire which may also have caused bias to the results. Although specific arguments for different approaches for diagnostics and management would give more insight into the current clinical practice, these were not directly asked in this study as this study was chosen to be descriptive in order to give a clear overview in the disease management of PIPO patients. The present study did not seek to ascertain whether the PIPO diagnostic criteria were applied to individual patients in each of the centers. In the future, we are planning to gather data on individual patients and will also aim to study the implementation of the recommendations at individual patient level.

## CONCLUSIONS

We conclude that wide diversity exists in all aspects of PIPO diagnostics, management, and treatment strategies across ERNICA IF teams. Although this study gathered the ERNICA IF teams with most experience, the numbers of patients per center were low. This indicates the need for regional reference centers with specialized multidisciplinary IF teams. Continuous collaboration between specialized centers is essential for improving outcomes of PIPO. This study brings important information on the current status of PIPO diagnostic and management strategies among ERNICA IF teams. Furthermore, it provides a basis for collaborative studies to develop new up to date recommendations for management guidelines. In the future, this will lead to improvement of the quality of patient care and outcomes for this patient group.

## Supplementary Material


