# Supplementary material for: Pediatric Intestinal Pseudo-Obstruction: An International Survey on Diagnostic and Management Strategies in the European Reference Network for Rare Inherited and Congenital Anomalies Intestinal Failure Teams
Source: J Pediatr Gastroenterol Nutr. 2023 Apr 7;77(1):24–30. doi: 10.1097/MPG.0000000000003788 (PMC10697283; doi:10.1097/MPG.0000000000003788)
Supplement: Supplementary file 1 [file mpg-77-24-s001.pdf]

Survey on pediatric intestinal pseudo-obstruction (PIPO): epidemiology and management

- This online survey will be sent to all ERNICA IF group members

- In this survey we use the following definitions:
Definition of pediatric chronic intestinal pseudo obstruction (PIPO): a chronic (persistence for 2 months from birth or at least 6 months thereafter) inability of the gastrointestinal tract to propel its contents mimicking mechanical obstruction, in the absence of any lesion occluding the gut occurring in children.

Definition of chronic intestinal pseudo-obstruction is a (CIPO): a chronic inability of the gastrointestinal tract to propel its contents mimicking mechanical obstruction, in the absence of any lesion occluding the gut occurring in adults.

Baseline data

- Country
- Institution
- Population that your institution covers (milj)
- Your profession: General pediatrician, Pediatric gastroenterologist, Adult gastroenterologist, Pediatric Surgeon, Adult Surgeon, Nutritionist, nurse, Parent, Other
- Is your institution taking care of patients with PIPO? Yes/no

Epidemiology

- Currently, how many patients with PIPO you have under active follow up in your institution? 0, 1-5. 6-10, 11-15, 16-20, 21-25, over 26
- Number of PIPO patients under 16y of age
- Are you following up both pediatric and adult patients with pseudo-obstruction (CIPO and PIPO)? Yes/No
- If no, what is the upper age limit of patients in your institution?
- On average, how many NEW PIPO patients per year you have in your institution?
- Are PIPO patients who have weaned off PN followed up in your institution?
- How many of the PIPO patients in your institution are currently dependent on (total or partial) parenteral nutrition?

Diagnosis and evaluation of PIPO

- Who makes the diagnosis of PIPO (choose all appropriate)? Pediatric gastroenterologist, Neonatologist/pediatrician, Surgeon, Multidisciplinary team, Other
- If multidisciplinary team approach is used for management of PIPO in your institution, the team includes (choose all appropriate): General pediatrician/adolescent medicine, Gastroenterologist, Surgeon, Nutritionist, Phychologist, Urologist, Geneticist/metabolic medicine specialist, Social worker, Nurse, Other
- Studies used for evaluation of PIPO: routinely used yes/no, study available yes/no
    - Is abdominal radiography used for PIPO diagnostics and/or evaluation?
    - Is small bowel follow-through study with water-soluble contrast used for PIPO diagnostics and/or evaluation?
    - Is entero-computed tomography (CT) study used for PIPO diagnostics and/or evaluation?

    - Is entero-magnetic resonance imaging (MRI) study used for PIPO diagnostics and/or evaluation?
    - Is multidetector-row helical CT study used for PIPO diagnostics and/or evaluation?
    - Is urinary tract ultrasound study used for PIPO diagnostics and/or evaluation?
    - Is excretory urogram study used for PIPO diagnostics and/or evaluation?
    - Are radio-opaque marker studies used for PIPO diagnostics and/or evaluation?
    - Is scintigraphy for measurement of gastric emptying used for PIPO diagnostics and/or evaluation?
    - Is H2 (C13) breath test for indirect measurement of gastric emptying used for PIPO diagnostics and/or evaluation?
    - Is scintigraphy for the measurement of small bowel and colon transit used for PIPO diagnostics and/or evaluation?
    - Is H2 breath test for the measurement of small bowel transit used for PIPO diagnostics and/or evaluation?
    - Is antroduodenal manometry used for PIPO diagnostics and/or evaluation?
    - Is esophageal manometry used for PIPO diagnostics and/or evaluation?
    - Is colonic manometry used for PIPO diagnosctics and/or evaluation?
    - Is anorectal manometry used for PIPO diagnostics and/or evaluation?
    - Is upper gastrointestinal endoscopy used for PIPO diagnostics and/or evaluation?
    - Is colonoscopy used for PIPO diagnostics and/or evaluation?
    - Full-thickness intestinal biopsy should be obtained for histopathological analysis for the diagnosis of PIPO (choose all appropriate) When therapeutic surgery is performed / for diagnostic intent alone / no biopsies are needed
- Laboratory tests routinely used (choose all appropriate)
    - Complete blood count
    - Electrolytes
    - Albumin
    - Renal and liver function tests
    - Inflammatory indices (ESR and CRP)
    - Coeliac serology [Tissue transglutaminase (tTG), anti-endomysial (EmA) IgA]
    - Thyroid function (TSH, related free hormone fractions)
    - Fasting cortisol
    - Metabolic panel (ammonia, lactate, urinary organic acids)
    - Cytomegalovirus
    - Epstein-Barr virus
    - Serum glucose and HbA1C
    - Connective tissue and skeletal muscle disorders (ANA, anti-ds-DNA, SCL-70, creatinine phosphokinase, aldolase)
    - Urinary porphyrins
    - Other
- Is genetic testing used for PIPO diagnostics in your institution? Routinely used yes/no
- If genetic testing is used for PIPO diagnostics, is there a routine genetic panel for PIPO?

Nutritional management

- The nutritional management strategy (choose all appropriate): favors oral feeding, favors use of gastrostomy or jejunostomy feeds, favors bolus feeding, favors continuous feeding, favors optimizing enteral feeding without compromising intestinal function, Other
- Who is mainly guiding the enteral nutrition? Nutritionist, Gastroenterologist, Surgeon, Multidisciplinary team, Other
- Home parenteral nutrition
    - Available yes/no
    - Routinely used yes/no

Medical management

- What prokinetic medication is used for PIPO in your institution? routinely tested to all PIPO patient, used after individual medical planning, never used
    - Amoxicillin/clavulanate
    - Azitromycin
    - Bethanecol
    - Cisapride
    - Domperidone
    - Erythromycine
    - Metoclopramide
    - Neostigmine
    - Octreotide
    - Prucalopride
    - Pyristigmine bromide

Surgical management

- Venting/feeding gastrostomy and/or jejunostomy is considered for all PIPO patients? yes/no
- Decompressing ileostomy is considered for all patients with PIPO on parenteral nutrition? yes/no
- The number of surgical interventions is minimized (choose all appropriate): in order to avoid potential complications (adhesions formation, prolonged paralytic ileus, post-surgery), for diagnostic uncertainty regarding pseudo-obstructive or true occlusive nature of future obstructive episodes, other
- Small bowel resection is (choose all appropriate): avoided to prevent occurrence of short bowel syndrome, avoided to prevent occurrence of intestinal failure associated liver disease, avoided to prevent reduction of abdominal domain in the view of potential future intestinal transplantation, not avoided
- Is intestinal transplantation available in your institution? yes/no
- Intestinal transplantation is considered as a therapeutic option for PIPO in case of (choose all appropriate): life-threatening complications of parenteral nutrition (IFALD, loss of central line access), poor quality of life with high risk of morbidity and mortality (frequent pseudo-obstructive episodes necessitating repeated hospitalizations, difficult fluid-electrolyte imbalances, repeated septic episodes), poor quality of life, other
